# Dietary Supplement of Grape Wastes Enhances Honeybee Immune System and Reduces Deformed Wing Virus (DWV) Load

**DOI:** 10.3390/antiox12010054

**Published:** 2022-12-27

**Authors:** Guillermo Pascual, Diego Silva, Marisol Vargas, Mario Aranda, Juan Antonio Cañumir, María Dolores López

**Affiliations:** 1 Departamento de Producción Vegetal, Facultad de Agronomía, Universidad de Concepción, Vicente Méndez #595, Chillán 3780000, Chile; 2 Laboratorio de Investigación en Fármacos y Alimentos, Departamento de Farmacia, Facultad de Química y de Farmacia, Pontificia Universidad Católica de Chile, Vicuña Mackenna 4860, Macul, Santiago 7810000, Chile; 3 Laboratorio de Bioprocesos, Departamento de Agroindustría, Facultad de Ingenería Agrícola, Universidad de Concepción, Vicente Méndez #595, Chillán 3780000, Chile

**Keywords:** winemaking industry, anthocyanins, *Apis mellifera*, antiviral activity, agro-sustainability, food waste valorization

## Abstract

Ingredients rich in phenolic compounds and antioxidants of winemaking wastes, which play an important role in the prevention of various diseases and the control of viruses, are being explored. Currently, there is a concern about honeybee population loss, with deformed wing virus (DWV) being the most common virus infecting apiaries and one of the main causes of honeybee decline. Hence, the effect of grape pomace powder (GPP) as a dietary supplement to enhance the immune system of honeybees affected by DWV was evaluated. The characteristics of the ingredient GPP, obtained by spray-drying, revealed a high anthocyanin content (1102.45 mg 100 g^−1^), and it was applied at doses of 0.5, 1, 2.5 and 5% as a dietary supplement for bees infected by DWV. The results showed that the GPP treatments strengthened the immune response of honeybees against DWV. Moreover, the expression of the Relish gene was significantly higher in bees fed with GPP compared to the infected control. This study, which is framed in the search of food waste valorization for environmental sustainability, proves the feasibility of using grape wastes as dietary supplements for pollinators, and provides knowledge of the influence of polyphenols on the expression profiles of immune-related genes in honeybees.

## 1. Introduction

Integrated solutions to climate change, resource scarcity, environmental degradation, food waste management, and an increasing demand for food are essential for the future of food and agriculture. In this sense, new products and strategies based on the reuse of environmentally sustainable materials are increasingly emerging, while technologies can broaden the scope of intervention, facilitating and accelerating the development of waste-free food systems.

Wine production reached 260 mhL (excluding juices and musts) in 2020 [1], generating a large amount of wastes. Traditionally, grape byproducts have been used as fertilizers and/or biomass. In recent years, however, they have been proposed as an interesting source of bioactive compounds that could be used for several applications [2].

Pomace from red grapes, one of the main winemaking wastes, contains phenolic compounds such as anthocyanidins, catechins, proanthocyanidins, tannins and organic acids with proven antiviral activity [3]. For instance, it has been reported that compounds of wine and its byproducts can inactivate viruses such as herpes simplex (HSV) type 1 and enteric viruses [4,5,6]. In addition, several studies have focused on the mechanisms of action of these bioactive compounds, demonstrating that these molecules can inhibit adsorption, as well as virus binding and virus entry, integrase, RTase and protease. In addition, they act on the inhibition of replication of DNA and RNA polymerases as well as the formation of protein complexes [7].

Hence, opportunities to reduce food waste and integrate this approach to the entire supply chain and reap the beneficial properties of these bioactive compounds are required. In this sense, the use of new ingredients for pollinators, as proposed in this study, could be a potential alternative for food waste reduction. Similar results were found in a study where they pointed out that there was a response in the genes involved in detoxification in bees fed with sucrose solution enriched by phenolic compounds [8].

The honeybee (*Apis mellifera* L.) is an important species with a key role in agriculture as a pollinator of plant species. Honeybees are involved in enhancing the biodiversity of agricultural and non-agricultural systems [9]. Therefore, honeybee losses have become a matter of increasing concern worldwide. Deformed wing virus (DWV), which is a widespread virus associated with the spread of the *Varroa destructor* mite, is considered as one of the most critical factors contributing to bee losses at the global level [10]. The virus causes physical malformations in wings, abdominal swelling, paralysis, and behavioral disturbances in bees. In addition, studies have reported that DWV infection can alter the molecular mechanisms of learning, affecting memorization ability and disrupting the central and peripheral nervous systems [11]. DWV transmission follows several routes, with varying pathogen loads and virulence. This transmission of the virus (DWV) in *A. mellifera* colonies can occur in two ways. On the one hand, it can be transmitted vertically (from the queen or drone to the offspring). On the other hand, it can be transmitted horizontally through either shared food resources or via trophallaxis [12]. Consequently, viral diseases have demonstrated a negative impact on food security, wildlife and economic stability [13].

Currently, beekeepers have several tools to control the vector of DWV, the Varroa mite. Among the measures, the use of synthetic chemical acaricides or natural repellents, such as essential oils, organic acids and a combination of the use of management practices are included, although they are not sufficiently effective [14]. Moreover, treatments for DWV are focused on vector control, but there is no curative treatment for colonies with high viral loads.

Biotic and abiotic factors are key contributors to colony health and survival (e.g., the diet of the colony), constituting alternatives to controlling DWV. It has been demonstrated that greater DWV levels are related to the consumption of a poor-quality diet (e.g., sugar water). Nevertheless, bees fed higher-quality food, for instance pollen-containing diets, present low DWV levels [15]. Phytochemicals, particularly phenolics, terpenoids and alkaloids, present antimicrobial activity [16] and even protect against microbial alterations in bees. Recently, researchers have described the antiviral activity of mycelium extracts, specifically of multiple polypore fungal species. In fact, [17] they found that the health of honeybees was enhanced after feeding water was enriched with a fungi source and their antimicrobial compounds, considering this situation to be self-medication. In addition, it has been reported that levels of DWV decreased up to 500-fold when a syrup of sugar and thymol, a monoterpene isolated from thyme, was used as food for young bees and it was released into field colonies [18]. With respect to phenolic compounds, the methanolic extracts from Chilean native plant leaves such as *Ugni molinae*, *Gevuina avellana* and *Aristotelia chilensis* were evaluated on the load of *N. ceranae*, seeking biological activity [19]. The authors concluded that leaf extracts, with high concentrations of rutin and myricetin, and propolis with high concentrations of galangin and pinocembrin, presented antiparasitic and antimicrobial activity. In addition, they proved an improved survival of bees and a decreased load of *N. ceranae* in infected bees and described some of the mechanisms involved in the enhanced immune system response of honeybees by the action of the phytochemicals.

Honeybees are endowed with an RNA interference (RNAi) molecular machinery and a eukaryotic antiviral immune system [20]. The main pathway of the antiviral defense mechanism in insects is mediated by the small interfering RNA (siRNA) belonging to a post-transcriptional, sequence-specific, gene silencing mechanism [21]. This RNAi could be induced by feeding or injecting dsRNA as an alternative to prevent gene expression and, consequently, block the replication of RNA viruses, including DWV [22,23]. As with other insects, honeybee antiviral responses also include apoptosis, eicosanoid biosynthesis, autophagy, melanization and endocytosis. Furthermore, Toll, NF-κB (Nuclear Factor κB), JAK/STAT (Janus Kinase/Signal Transducer and Activator of Transcription), JNK (c-Jun N-terminal kinase), and MAPK (Mitogen-Activated Protein Kinases) pathways are found [24].

Consequently, grape wastes, specifically grape pomace, which is rich in phenolic compounds, could be formulated as a supplement, enhancing the honeybee immune system. In order to protect bioactive compounds obtained from grape waste and avoid their degradation over time, encapsulation techniques have been developed to ensure their stability. Among them, spray drying has been used widely in the encapsulation to large-scale production since it is economical and adaptable, while it makes a product of excellent quality. The process generates stability and protection to the bioactive compounds by acting as a physical barrier and allowing for slow release. Concerning the loss of bioactive content, the high temperature in the process is the main issue, and thus the process needs to be optimized, while the properties and bioactivity of the final ingredient should be studied [25]. Studies carried out with Tintorera have shown that the use of this technique preserves anthocyanin content by maintaining the formulation for four weeks [26]. The authors demonstrated that after spray drying, the ingredient had an adequate anthocyanin encapsulation efficiency, resulting in increased stability and bioaccessibility.

The use of environmentally friendly alternatives to help mitigate phytosanitary problems in apicultural systems and the reuse of winemaking waste is worthy for environmental sustainability and food security. Therefore, the objective of this research was to evaluate the effects of grape pomace powder as a dietary supplement to enhance the immune system of honeybees affected by DWV.

## 2. Materials and Methods

### 2.1. Material

Grape pomace var. Tintorera was collected in the Itata Valley, Ñuble Region, (36°37′6.96″ S, 72°19′30.81″ W) in the 2021 season. The pomace was frozen and transported to the BIOINVE (Bioactive Compounds and Ingredients from Plants Laboratory, Faculty of Agronomy, University of Concepción, Concepción, Chile) and kept at −80 °C until analysis.

### 2.2. Preparation of the Microencapsulated Supplement by Spray Drying and Characterization of the Grape Pomace Powder (GPP)

Batchs of 500 g of grape pomace were mixed with 96% ethanol (*w*/*v*) following the methodology described by Romero-Román et al. [27]. Maltodextrin DE-10 at 20% (*w*/*v*) was used as wall material and was acquired from the Sigma Aldrich Chemical Co. (MilliporeSigma, St. Louis, MO, USA). The mini spray-dryer B-290 (Büchi, Flawil, Switzerland) was fed with the aforementioned solution and was homogenized by constant agitation at room temperature and at a flow rate of 4 mL min^−1^. The inlet temperature was maintained at 120 °C, whereas the outlet air temperature was 80 °C (Table S1). The powder obtained was collected in an opaque, air-tight container at 4 °C for additional analysis. The morphological characterization of the grape pomace powder (GPP) was carried out by Scanning Electron Microscopy (SEM) (FEI-Inspect S50, FEI Company, Hillsboro, OR, USA). GPP samples were prepared and attached to double-sided adhesive tape mounted on SEM stubs, covered with a gold layer (9 nm) under vacuum SEM working at 5 kV, with 10,003 and 50,003 magnifications. Finally, loading capacity (quantity of total phenolic compounds per 100 grams of powder), entrapment efficiency (g total phenolic compounds encapsulated 100 g^−1^ phenolic compounds from pomace added) and powder recovery (ratio between the quantities of powder versus the initial mass solids) were calculated (Table S2). Total phenolic compounds (g) were obtained by HPLC-DAD at 280, 320, 360 and 520 nm similar to that described in Section 2.3.

### 2.3. Characterization and Quantification of Phenolic Compounds and Antioxidant Properties from Grape Pomace Powder

The extraction and chemical characterization of the GPP was performed according to the methodology described by López-Belchí et al. [26]. Phenolic compounds were extracted and quantified by a Hitachi HPLC-DAD system (Hitachi technologies, MERCK, Darmstadt, Germany) using the same chromatographic conditions described by López-Belchí et al. [26], recording chromatograms at 280, 320, 360 and 520 nm. Some external standards were used for quantification, such as gallic acid (99%), (+)-catechin (99%), epigallocatechin gallate (95%), 3-hydroxytyrosol (99%) and tyrosol (99) at 280 nm, chlorogenic acid (97%), caffeic acid (99%), coumaric acid (99%), ferulic acid (99%) and *trans*-resveratrol (99%) at 320 nm, quercetin hydrate (95%), quercetin 3-*O*-glucoside (90%), myricetin 3-*O*-glucoside (96%), kaempferol 3-*O*-glucoside (99%) at 360 nm, malvidin 3-*O*-glucoside (95%) and cyanidin 3-*O*-glucoside (99%) at 520 nm, (Sigma-Aldrich, St. Louis, MO, USA). All of the solvents used in the extractions were of analytical grade and were obtained from Merck (Darmstadt, Germany). The results were expressed in mg 100 g^−1^ DW.

The antioxidant activity was determined by DPPH* (2,2-diphenyl-1-picrylhydrazyl) and ORAC (oxygen radical absorbance capacity) assays [26,28,29]. Briefly, the antioxidant capacity for ORAC-FL was estimated by measuring the changes in fluorescence after 120 min of reaction with the radical, whereas DPPH* was evaluated by determining the variation in absorbance at 515 nm after 30 min of reaction with the radical. Both assays were performed using a 96-well micro in a Synergy H1 hybrid multi-mode microplate reader (Biotek, Winooski, VT, USA), and the results were expressed as μmol Trolox 100 g^−1^ DW. Six replicates were accomplished for each analysis.

### 2.4. Inoculum DWV-A Preparation

Deformed wing virus variant A (DWV-A) was isolated and obtained from infected colonies following the methodology described by Gusachenko et al. [30]. To carry out this experiment, a group of 20 honeybees were homogenized in a stomacher bag with phosphate buffered saline (1X PBS) for 90 s at high speed in a stomacher 80 lab blender (Seward, London, UK). Samples were centrifuged twice at 1500× *g* for 10 min at 4 °C, followed by 10,000× *g* for 10 min at the same temperature. Afterwards, the supernatant was filtered and purified with a 0.22 μm filter (PES, Merck Millipore, Darmstadt, Germany). Subsequently, to perform the extraction of RNA, the synthesis of cDNA and the subsequent quantification of the viral load of the inoculum, 200 μL of the supernatant was used following the methodology described in Section 2.6. The rest of the extracted inoculum was stored at −80 °C until it was analyzed.

### 2.5. Bee Inoculation and Experimental Design

*A. mellifera* adults were acquired from the experimental apiary located in El Nogal Experimental Station (36°35′58.25″ S−72°04′51.77″ W), University of Concepción, Chillán, Chile. Previous to the assay, the health status of the apiary was determined by identifying the viral level in each colony (*n* = 54 beehives). The pathogen level was established by molecular techniques according to Vargas, et al. [31]. Brood combs with capped worker bees were removed from colonies with low DWV load (1.0 × 10^2^ copy number per bee). In addition, no colonies were detected to be DWV-free. The brood combs were maintained under controlled conditions in a rearing room (30 °C ± 1; 60% ± 3 RH). Afterwards, newly emerged worker bees (24 h old) were carefully collected from the brood combs and randomly confined in plastic cages (base = 8 cm diameter, mouth = 10 cm diameter, and height = 15 cm). Bees were inoculated orally with 5 μL of a viral suspension (1.0 × 10^9^ copy number per bee) in a 50% sucrose solution according to the methodology described by Porrini et al. [32]. The bees that did not consume the total amount of viral inoculum were discarded from the assay. Next, four treatments consisting of different doses of GPP (0.5%, 1%, 2.5% and 5%) dissolved in a sugar syrup at 50% weight/volume ratio were used ad libitum. Two control treatments without GPP were also included, a non-inoculated (N-DWV) control and an inoculated (I-DWV) control (see Klapan-Meier survival curves in Supplementary Material Figure S1). For all of the GPP treatments and controls, four replicates were carried out with 80 worker bees each. A dietary regimen was established for the treatments with the GPP. The regimen consisted of 24 h for the bioactive supplement and 48 h for the sugar syrup, both *ad libitum*, starting on day 0 and alternating until day 14 of the experiment, where the maximum viral load occurred as a result of viral replication [33]. In addition, all cages contained 3 g of pollen substitute. Five bees were collected per cage, starting on the day of virus inoculation according to the dietary regimen described. Finally, 11 collections were obtained during the whole assay, 17 days post-inoculation (20-day-old bees).

### 2.6. RNA Extraction and cDNA Synthesis

Total RNA was extracted following the methodology of Riveros et al. [34]. Briefly, five bees were collected by treatment, replicate and sampling time. They were ground in a PBS-buffered saline solution (5 mL) and 200 µL of this macerate was collected. Subsequently, 500 µL of TrizolTM (Invitrogen, Thermo Fisher Scientific, Waltham, MA, USA) were added and RNA extraction was carried out using an E.Z.N.A. ^®^Total RNA Kit I (Omega Bio-Tek, Norcross, GA, USA). In addition, RNA quality and yield were determined using a spectrometer (Infinite 200 PRO NanoQuant, Tecan Group, Männedorf, Switzerland). The extracted RNA was subsequently used for first-strand cDNA synthesis using the M-MLV reverse transcriptase enzyme (Invitrogen, Life Technologies, Carlsbad, CA, USA) following the manufacturer’s instructions.

### 2.7. Real-Time PCR Quantification (qPCR)

Viral load and expression of defense genes was quantified by using specific primers (Table 1). The PCR reaction was carried out using 1× of KAPA SYBR FAST Universal 2× qPCR Master Mix (Kapa Biosystems, Wilmington, MA, USA) according to the manufacturer’s instructions. Samples were added to a reaction volume of 15 µL, including 20 ng of cDNA, 530 nM of each primer, and sterile filtered molecular-grade water to reach 15 µL. The conditions used for the thermal reaction were 96 °C for 10 min, followed by 40 cycles at 95 °C for 15 s, 60 °C for 15 s, and 72 °C for 30 s. Real-time PCR assays were performed on a Stratagene Mx3000P thermal cycler (Agilent Technologies, Santa Clara, CA, USA), and the data were analyzed using MxPro software (Stratagene, Agilent Technologies, Santa Clara, CA, USA). The relative expression of each gene was calculated after normalization with an endogen gene (β-actin) as defined by Pfaffl [35]. The viral load was analyzed by the absolute quantification of DWV-A.

A standard curve by means of a purified PCR product Wizard^®^ VR SV gel and PCR clean-up system (Promega, Madison, WI, USA), belonging to the viral target sequence, was utilized [34]. Purified amplicon was quantified by using spectrophotometry (EpochTM Microplate Spectrophotometer, BioTek, Winooski, VT, USA) and a copy number according to Wu, et al. [40] was calculated.

Linear standard curves (95–100% efficiency) were applied using a serial dilution (1.0 *×* 10^1^ to 1.0 *×* 10^9^) of viral copy numbers of purified cDNA. Subsequently, the cycle threshold (Ct) values were plotted against copy number values (log_10_). The sample copy numbers were estimated using the Ct values and comparisons with the linear equation of the standard curve and normalizing values of the housekeeping β-actin gene were made [36]. Data were expressed as the copy number of DWV per bee by considering the dilutions that were achieved in the cDNA synthesis and qPCR reaction.

### 2.8. Data Analysis

The effectiveness and achievability of the ingredient based on grape pomace was established based on the response of the honeybee immune system. To analyze gene expression levels, a one-way analysis of variance (ANOVA), followed by Tukey’s post hoc test for significance was used through R software version 4.0.3. The viral load was analyzed using a Tukey’s test. A *p* < 0.05 indicated a statistically significant result.

## 3. Results and Discussion

### 3.1. Characterization of the Grape Pomace Dietary Supplement

**Figure 1:**
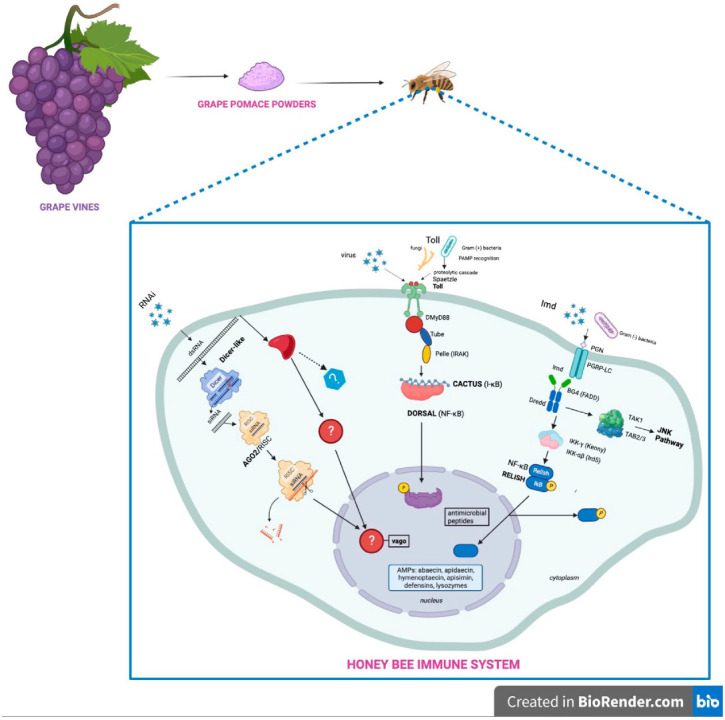
Workflow of the production of GPP as a dietary supplement to strengthen the honeybee immune system.

Diet plays a fundamental role in providing strong and healthy honeybee colonies [41]. In fact, there is evidence that the capacity of the colony to face both biotic and abiotic stressors can be enhanced by providing strong bees through a convenient supply of nutrients [42], activating the most important protective physiological system of all organisms, the immune system. The workflow presented in this study follows the scheme depicted in Figure 1.

Grape pomace [43] and GPP obtained by spray drying were chromatographically analyzed (Figure S2). It is important to point out that the percentage of wall material used (20% maltodextrin) had been previously studied, with satisfactory results [26]. Different phenolic compounds presented a high content, such as syringic acid (153 and 75 mg 100 g^−1^ DW for GP and GPP, respectively), and catechin (67 and 43 mg 100 g^−1^ DW for GP and GPP, respectively) (Table 2). However, the greater phenolic content was found in the anthocyanin group, reaching a total of 1873 mg 100 g^−1^ DW for GP and 1102 mg 100 g^−1^ DW for GPP. Additionally, ten anthocyanins, reaching high content, were identified by HPLC-DAD, and the resulting concentration agreed with those reported by Wu et al. [43]. The main anthocyanins quantified from GP raw material and GPP were malvidin 3-*O*-hexoside and petunidin 3-*O*-hexoside, while content losses of the compounds ranged between 36 and 53%, with an average of 41% of the GPP with respect to the GP raw material. It is important to point out that despite the loss of phenolic compounds due to the drying process (see GPP), the final content was still high, and the phenolic compound and anthocyanin compound concentrations were found to be within the range reported for dry grape pomace (1598.57 mg 100 g^−1^ gallic acid equivalents and between 730.7–1850.3 mg 100 g^−1^ malvidin 3-glucoside equivalents, respectively) by Yeler et al. [44]. Furthermore, it is important to mention their high anthocyanin content, particularly if they are compared with food with great value of phenolic compounds such as blueberry (around 840 mg 100 g^−1^ DW) [45] or elderberry (range 800–1000 mg 100 g^−1^ DW) [46], making, therefore, this ingredient a source of bioactive compounds to supplement the diet of bees.

It is demonstrated that spray-drying treatments would be adequate for the treatment of waste with a high content of bioactive compounds, such as the case of grape pomace, since it is easy to handle, cheap, scalable, and the ingredient obtained would remain enriched in compounds of interest. Furthermore, it is worthy of note that this research was based only on the polyphenolic composition of grape pomace; however, grape skins represent a more complex matrix. In fact, other compounds or nutrients such as vitamins can contribute substantially to the biological activity [47].

The antioxidant activity was determined by using two assays, 2,2-diphenyl-1-picrylhydrazyl (DPPH*) and oxygen radical absorbance capacity [28]. Assays were carried out and measured for the raw material, GP, and for the ingredients obtained after the spray-drying process (GPP), obtaining high antioxidant activity for GPP (60% of the activity in the ORAC assay compared to GP) (Table 3).

**Figure 2:**
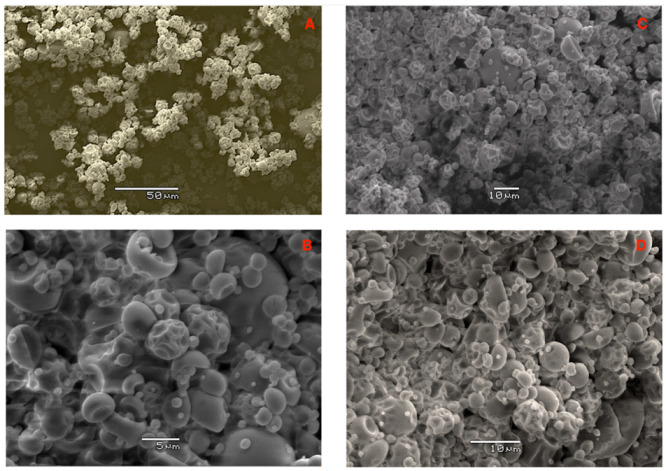
SEM images of the spray-dried grape pomace powder encapsulated with 20% maltodextrin for the spray-drying condition of 120 °C inlet air temperature. Different scales are shown: 50 μm (**A**); 5 μm (**B**); and 10 μm (**C**,**D**).

In relation to the antioxidant activity, despite the heat treatment, the activity shown was adequate. Fruits with high antioxidant potential such as blueberries presented similar values to GPP, and even lower values in DPPH (530 μmol TE 100 g^−1^ DW) and ORAC (2300 μmol TE 100 g^−1^ DW) [48]. After encapsulation, micrographs of the formulation were visualized and analyzed (Figure 2). SEM images showed a spherical shape, although most of its surface was depressed. The micrographs also confirmed that the wall material utilized, maltodextrin, worked effectively and efficiently [49]. It was also possible to achieve an estimation of the particle size of the anthocyanin-rich grape pomace coatings by this visualization. Due to agglomeration, some particles showed a slightly rough surface. Particle sizes were stable (average 7 μm).

Grape pomace and ingredients based on it has a high added value due to its high content of bioactive compounds and its wide variety of applications. Therefore, given the great number of wastes generated by the wine industry, the reintroduction of new high-value ingredients would represent a good alternative to address food waste management.

### 3.2. DWV Load after Feeding with a Grape Pomace Supplement

**Figure 3:**
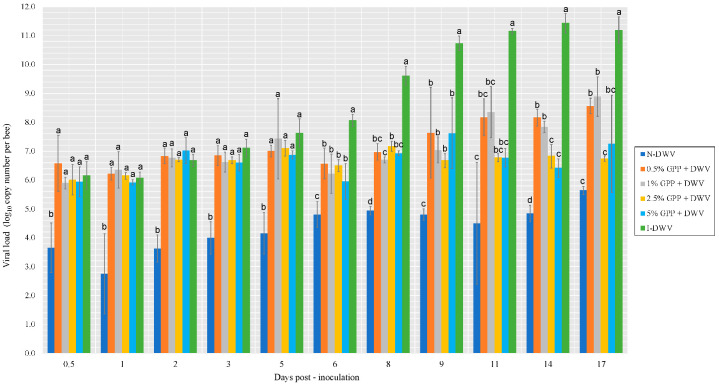
DWV loads in worker honeybees recorded in days post-inoculation throughout the assay. Grape pomace powder (GPP) concentrations (0.5, 1, 2.5 and 5%), and inoculated control (I-DWV) and non-inoculated control (N-DWV) are shown in different colors. Different letters indicate significant differences according to a Tukey’s test (*p* < 0.005).

The relationship between honeybee viral load and the provision of a supplementary diet rich in bioactive compounds was studied, while the transcriptional profiles of multiple separate (though inextricably coupled) immune pathways were determined and analyzed with respect to DWV. With respect to the honeybee viral loads, I-DWV presented similar viral loads to the GPP treatments on 0.5 day post-inoculation, while N-DWV showed significantly lower differences that did not exceed a viral load of 6.0 (1.0 × 10^6^ copy number per bee) (Figure 3).

Viral load varied significantly between bees that were inoculated with DWV and supplemented with GPP (doses of 0.5, 1, 2.5 and 5%) and those inoculated (I-DWV) (Figure 3). These differences were observed for each collection day, from day 6 post-inoculation until the end of the assay. All of the treatments containing GPP decreased in viral load, showing significant differences with respect to I-DWV and N-DWV. For example, the viral load recorded on day 6 post-inoculation was 30% lower in bees fed with the GPP supplement at 5% (1.0 × 10^6^ copy number per bee) compared to the I-DWV control (1.0 × 10^11^ copy number per bee), with significant differences between the treatments. On day 11 post-inoculation, the viral load decreased by 30% with the 0.5 and 1% GPP treatments, while the 2.5 and 5% GPP treatments resulted in reductions of 40% with respect to I-DWV. These results are in agreement with Felicioli, et al. [50], who tested an artificial diet capable of stimulating the immune system in bees, and reported that the provision of 1,3-1,6 β-glucans to honeybees infected by DWV reduced or maintained the DWV viral load compared with the control group.

### 3.3. Response in Honeybee Gene Expression after Feeding with a Grape Pomace Supplement

Studies on the transcriptional level in honeybees involve uncharacterized genes/pathways in antiviral responses [22]. In this sense, the roles of genes in the Toll, Imd, Jak-STAT, JNK, and RNAi pathways are the best characterized. In the present study, colonies with very low levels of DWV were used in the absence of *Varroa* or other pathogens according to the strategy described in other comparable studies [33,51,52].

RNAi is the main antiviral mechanism in insects [53]; therefore, honeybees were expected to mount an RNAi response when inoculated with DWV. In fact, the RNAi-pathway is initiated by the Dm Dicer-2 cleavage of viral dsRNA into 21–22 bp siRNAs. It is important to point out that Am Dicer-like share 30% aa identity with Dm Dicer-2. In addition, the siRNAs are loaded into AGO2 (Argonaute-2), the catalytic component of the RISC (RNA Induced Silencing Complex) [54]. In this situation, a single strand of the siRNA is retained in the RISC and used to specifically target cognate the viral genome sequences for cleavage. In addition, Dm Dicer-2 works as a dsRNA sensor that mediates a signal transduction cascade resulting in increased expression of Dm Vago and suppression of viral replication. Am Dicer-like may serve as a dsRNA sensor, and honeybees have a Vago orthologue, which is upregulated in DWV-infected honeybees [22].

**Figure 4:**
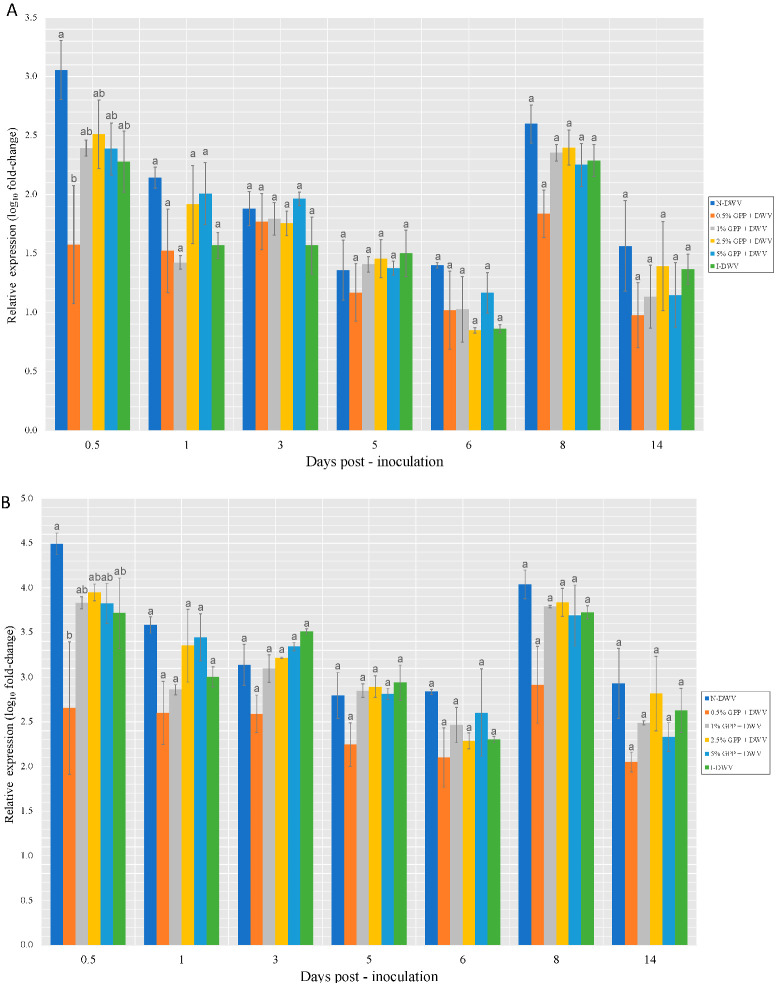
Expression level of Dicer (**A**) and Argo-2 (**B**) genes involved in the RNAi-pathway in worker honeybees per collection throughout the assay. Grape pomace powder (GPP) concentrations (0.5, 1, 2.5 and 5%), inoculated control (I-DWV), and non-inoculated control (N-DWV) are shown in different colors. Different letters indicate significant differences according to Tukey’s test (*p* < 0.005).

Even though no significant differences in Dicer and Argo-2 expression were found between the treatments, peaks of expression were observed on collection days 1 and 8 for low and medium doses (0.5, 1, 2.5%) which showed higher gene expression (Figure 4).

The relationship between the *Varroa* parasite and DWV was studied, showing that the expression of both Dicer and Argonaute-2 increased in parallel with DWV viral loads in pupae over time. However, in this study it was observed that Dicer was the only gene that was up-regulated in DWV-injected pupae compared to the two control groups at all time points [55]. In our research, Dicer showed jumps in expression throughout the assay (Figure 4A).

**Figure 5:**
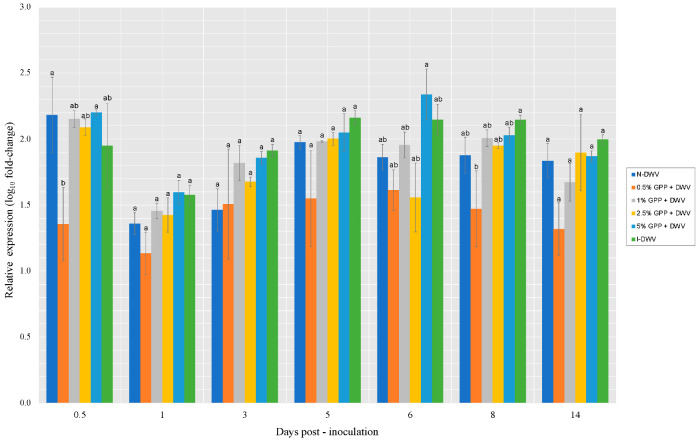
Expression level of *Vago* gene in worker honeybees per collection throughout the assay. Grape pomace powder (GPP) concentrations (0.5, 1, 2.5 and 5%), inoculated control (I-DWV) and non-inoculated control (N-DWV) are shown in different colors. Different letters indicate significant differences according to Tukey’s test (*p* < 0.005).

Although the quantification of the transcript expression of the Vago gene revealed some significant differences, it is important to highlight the low expression observed for the lowest concentration (0.5%) throughout the assay compared to the controls and the other GPP treatments (Figure 5). A direct relationship between the expression of these two genes and the expression of Vago was also found. In *B. terrestris*, Vago limits viral infection in fat bodies in a Dicer-dependent manner [54].

The principal insect innate immune response for the detecting and inhibiting virus replication in insects is through RNA interference (RNAi) via double stranded RNA (dsRNA) that is a sequence specific post-transcriptional gene-regulator [53]. Hence, the activation of this pathway in bees increases the expression of the Vago gene, an orthologue also found in *Drosophila*, and a suppressor viral replication [56].

The latest data suggest that the Toll and Imd pathways similarly contribute to defense against viral pathogens [50]. In the Toll pathway case, it is generally associated with fungi and gram-positive bacteria, and, on the other hand, the Imd pathway is associated with gram-negative bacteria [57]. Dorsal (Toll), and Relish (Imd) are both NF-κB family transcription factors that are involved in the production of antimicrobial peptides (AMPs) in fat bodies, a tissue analogous to the mammalian liver. It has been previously suggested that the Toll pathway, particularly the expression of NF-κB factor Dorsal-1A, is a strategic component regulating the immune response of honeybees against DWV [55,58].

**Figure 6:**
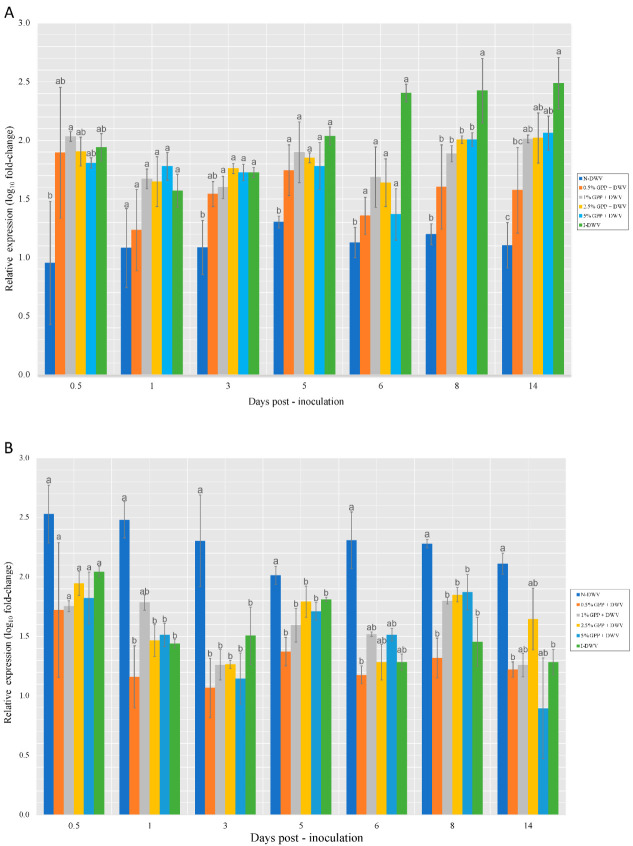
Expression level of *Cactus* (**A**) and *Dorsal* (**B**) genes involved in Toll pathway worker honeybees per collection throughout the assay. Grape pomace powder (GPP) concentrations (0.5, 1, 2.5 and 5%), inoculated control (I-DWV), and non-inoculated control (N-DWV). Different letters indicate significant differences according to a Tukey’s test (*p* < 0.005).

Cactus is an NF-κB inhibitor that is degraded to allow the nuclear translocation of Dorsal [59]. An increased cactus expression in DWV infected adult bees parasitized by *V. destructor* has been associated with the down-regulation of Dorsal [60]. This is consistent with the results of the present study, where significant differences were observed for Cactus and Dorsal in both control and GPP treatments (Figure 6), while the expression of Cactus silenced the expression of Dorsal mainly in the non-inoculated control (N-DWV).

All of the GPP treatments at different concentrations showed an intermediate expression with respect to N-DWV and I-DWV control treatments, with some exceptions. This clearly indicates that the dietary supplementation based on GPP influences the expression of Toll pathway genes.

The immune-deficiency signaling pathway (Imd) in bees and flies is activated by the Relish transcription factor (homologue to NF-κB transcription factor) [56].

In another insect, such as Drosophila, the immune pathways are strongly regulated in order to balance the immune responses. Therefore, the increased expression of pathway inhibitors, such as Cactus, does not necessarily indicate complete or continuous repression of the pathway (e.g., Toll) [22]. Relish is a transcription factor in the Imd pathway, which, upon cleavage, leads to the production of the antimicrobial peptides (AMPs) abaecin and hymenoptaecin [55].

**Figure 7:**
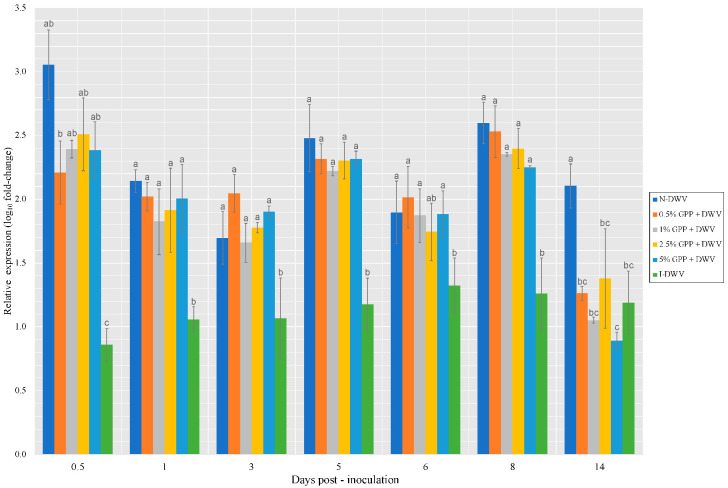
Expression level of Relish gene involved in the Imd pathway in worker honeybees per collection throughout the assay. Grape pomace powder (GPP) concentrations (0.5, 1, 2.5 and 5%), inoculated control (I-DWV), and non-inoculated control (N-DWV). Different letters indicate significant differences according to a Tukey’s test (*p* < 0.005).

In the present study, the quantification of the Relish gene (Figure 7) showed significant differences between the treatments with respect to the inoculated control (I-DWV) throughout the assay, showing a higher expression than I-DWV and maintaining a gene expression similar to N-DWV (before 14 days). There is evidence that both the Toll and Imd pathways defend against viral invasion in insects [61,62].

All of the treatments used with GPP presented differences from the control. However, this was not observed in a dose-dependent manner. The results showed that the GPP dietary supplement inhibited DWV infection in DWV-infected bees. This indicates that the bioactive compounds found in grape pomace strengthen the immune system by upregulating the expression of genes involved in pathways known to influence immune responses (RNAi, Toll and Imd), protecting honeybees from external stress caused by viral infections. A study on bees infected with DWV conducted by Lu, et al. [62] demonstrated that caffeine affected bee response against pathogens and the expression profiles of genes involved in the immune response. They showed that caffeine can increase the expression of genes involved in immunity and reduce the copy number of the virus, indicating that it has the potential to enhance to capacity of bees to fight against viral infection. The use of natural ingredients, such as antioxidant compounds, has been a great solution and benefit for the generation of new multi-targeted antiviral compounds, which can influence the viral life cycle and host proteins [63].

Although the results are shown separately for the viral load and the expression of each gene (with some significant differences), an overall view shows that each gene is reflected in the DWV viral load (Figure 3), which is consistent with the gene expression, and representing a lower viral load, which suggests that the supplement had an effect by reducing the viral load.

## 4. Conclusions

Given the great amount of waste and by-products generated by food processing (e.g., the wine-making industry), waste management valorization is essential to ensuring environmental sustainability. The present study evaluated the effect of different doses (0.5, 1, 1.5, 2.5 and 5%) of grape pomace powder (GPP) supplement on the immune system of honeybees, specifically its capacity to control deformed wing virus (DWV). The loads of DWV were lower after feeding with the supplement enriched in phenolic compounds (in the GPP treatments) with respect to the DWV inoculated control (I-DWV). The gene expression determined for the Relish gene showed significant differences for all the GPP doses used with respect to I-DWV, while the treatments showed no significant differences compared to the non-inoculated control (N-DWV), except for the lowest dose (0.5%) on collection day 1. The results allow for the conclusion that the inclusion of encapsulated GPP in the diet of honeybees is a promising alternative to improve bee health because it decreases DWV loads and influences the expression of genes involved in their immune system pathways. This study provides knowledge of the influence of the phenolic compounds of grape pomace on the expression profiles of immune-related genes in honeybees, offering an alternative to waste management from wine production.

## Figures and Tables

**Table 1:** Nucleotide sequence of defense gene primers from *Apis mellifera* and deformed wing virus (DWV-A) used in qRT-PCR.

Primers	Sequences		Reference
DWV-A	F	TATCTTCATTAAAGCCACCTGGAA	[36]
	R	TTTCCTCATTAACTGTGTCGTTGAT	
β-Actin	F	ATGCCAACACTGTCCTTTCTGG	[36]
	R	GACCCACCAATCCATACGGA	
Relish	F	GCAGTGTTGAAGGAGCTGAA	[37]
	R	CCAATTCTGAAAAGCGTCCA	
Cactus	F	CACAAGATCTGGAGCAACGA	[37]
	R	GCATTCTTGAAGGAGGAACG	
Argo-2	F	ACCTGCTGAGTTATGCACAGT	[38]
	R	AGCCTTTAGAACTCTTGCTGGT	
Dicer	F	AGCAGTAGCTGATTGTGTGGA	[38]
	R	TGAAGGATGTGTAAACGCCTGT	
Dorsal	F	CTCATCGGAAGACATGACAGTGA	[38]
	R	TGAATTCAAAGCCAGTTCGAAAA	
Amel102	F	CAACTCCAGAATTGGAAATAGCA	[39]
	R	TTTGCAATAGGAAAAGCAGTTG	

**Table 2:** Phenolic compound content (mg 100 g^−1^ DW) identified in grape pomace and grape pomace powder (GPP) from Tintorera grapes.

Compounds	GP	GPP	Anthocyanin Content Losses (%)
Anthocyanins			
Cyanidin 3-*O*-hexoside	175.79 a	96.25 b	45
Peonidin 3-*O*-hexoside	40.34 a	18.87 b	53
Delphinidin 3-*O*-hexoside	180.90 a	108.03 b	40
Petunidin 3-*O*-hexoside	403.70 a	227.93 b	43
Malvidin 3-*O*-hexoside	682.34 a	420.44 b	38
Malvidin 3-*O*-(6-acetyl)-glucoside	42.09 a	26.35 b	37
Delphinidin 3,5-*O*-di-hexoside	38.26 a	18.42 b	51
Malvidin 3,5-*O*-di-hexoside	189.39 a	111.56 b	41
Petunidin 3,5-*O*-di-hexoside	34.21 a	21.65 b	36
Malvidin derivatives	86.26 a	53.14 b	38
Total identified	1873.29 a	1102.65 b	41
Hydroxybenzoic acids			
Gallic acid	6.02 a	3.09 b	49
Syringic acid	153.65 a	75.33 b	50
Total identified	159.67 a	78.42 b	49
Hydroxycinnamic acids			
Chlorogenic acid	0.47 a	0.20 b	57
Caftaric acid	0.79 a	0.31 b	60
Caffeic acid	2.94 a	0.89 b	69
p-coumaric acid	6.17 a	2.90 b	52
Ferulic acid	5.16 a	2.12 b	58
Total identified	15.53 a	6.42 b	58
Stilbene			
*Trans*-Resveratrol	4.05 a	1.88 b	53
Total identified	4.05 a	1.88 b	53
Flavanols			
(+)-Catechin	67.93 a	43.79 b	35
(−)-Epicatechin	19.57 a	8.57 b	56
(−)-Epigallocatechin gallate	0.82 a	0.43 b	47
Total identified	88.32 a	52.79 b	40
Flavonols			
Quercetin 3-glucoside	6.56 a	2.93 b	55
Kaempferol 3-glucoside	3.73 a	2.92 a	21
Myricetin derivatives	0.44 a	0.22 b	50
Quercetin derivatives	1.54 a	0.93 a	39
Total identified	12.27 a	7.00 b	42
Phenylethanoids			
Hydroxytyrosol	1.83 a	0.98 b	46
Tyrosol	1.68 a	0.73 b	56
Total identified	3.51 a	1.31 b	62

Different letters within the same row indicate significant differences at *p* < 0.05 in drying treatments according to Tukey’s test.

**Table 3:** Antioxidant capacity (DPPH* and ORAC) of grape pomace and grape pomace powder (GPP) from Tintorera grapes.

Sample	DPPH*	ORAC
GP	9340 a	8153 a
GPP	2988 b	4921 b

DPPH* (µmol Trolox 100 g^−1^ of sample DW; ORAC (µmol Trolox 100 g^−1^ of sample DW)). Different letters in the same column indicate significant differences at (*p* ≤ 0.05).

## Data Availability

Not applicable.

## Supplementary Materials

The following supporting information can be downloaded at: https://www.mdpi.com/article/10.3390/antiox12010054/s1, Figure S1: Klapan-Meiners survival curves for 4 doses of GPP (0.5%; 1%; 2.5%; 5%) in honeybees; Figure S2: Chromatograms of each detection wavelength for GP and GPP analyzed by HPLC-DAD at (a) 280 nm, (b) 320 nm and (c) 360 nm and (d) 520 nm; Table S1: Summary of conditions of the preparation of spray-drying; Table S2: Mean values for loading capacity, powder recovery and entrapment efficiency of powder based on grape pomace obtained by spray-drying.
