# Supplementary material for: Dietary Supplement of Grape Wastes Enhances Honeybee Immune System and Reduces Deformed Wing Virus (DWV) Load
Source: Antioxidants (Basel). 2022 Dec 27;12(1):54. doi: 10.3390/antiox12010054 (PMC9855144; doi:10.3390/antiox12010054)
Supplement: Supplementary file 1 [file antioxidants-12-00054-s001.zip › antioxidants-2060405-supplementary.pdf]

antioxidants

Article

Dietary supplement of grape wastes enhances honeybee immune system and reduces deformed wing virus (DWV) load

Guillermo Pascual [1], Diego Silva [1], Marisol Vargas [1], Mario Aranda [2], Juan Antonio Cañumir [3], María Dolores López [1,*]

[1] Departamento de Producción Vegetal, Facultad de Agronomía, Universidad de Concepción, Vicente Méndez #595, Chillán3780000, Chile
[2] Laboratorio de Investigación en Fármacos y Alimentos, Departamento de Farmacia, Facultad de Química y de Farmacia, Pontificia Universidad Católica de Chile, Vicuña Mackenna 4860, Macul, Santiago 7810000, Chile
[3] Laboratorio de Bioprocesos, Departamento de Agroindustría, Facultad de Ingenería Agrícola, Universidad de Concepción, Vicente Méndez #595, Chillán3780000, Chile; jcanumir@udec.cl
* Correspondence: mlopezb@udec.cl

Supplementary Materials

Figure S1. Klapan-Meiners survival curves for 4 doses of GPP (0.5 %; 1 %; 2.5 %; 5 %) in honeybees.

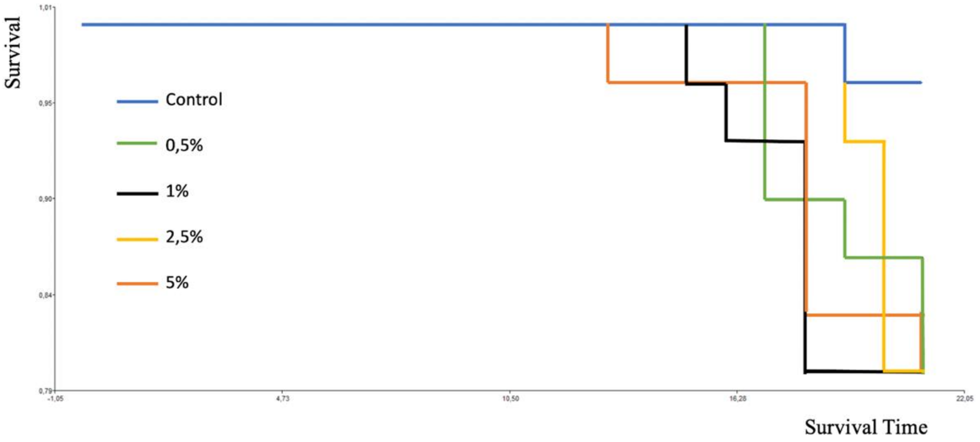

Chi-square treatment for log rank test=4.515 p=0.340820

Treatment	case	Time	Exposed	Deaths	Survival	E.E
0,50%	1	17	30	1	0,97	0,03
0,50%	6	18	29	5	0,80	0,07
0,50%	30	21	24	0	0,80	0,07
1,00%	1	15	30	1	0,97	0,03
1,00%	2	16	29	1	0,93	0,04
1,00%	3	17	28	1	0,90	0,05
1,00%	4	19	27	1	0,87	0,06
1,00%	6	20	26	2	0,80	0,07
1,00%	30	21	24	0	0,80	0,07
2,50%	3	19	30	2	0,93	0,04
2,50%	5	20	28	2	0,87	0,06
2,50%	30	21	26	1	0,83	0,07
5,00%	1	13	30	1	0,97	0,03
5,00%	3	17	29	1	0,93	0,04
5,00%	8	18	28	3	0,83	0,06
5,00%	30	21	25	1	0,80	0,07
control	1	19	30	1	0,97	0,03
control	30	21	29	0	0,97	0,03

Figure S2. Chromatograms of each detection wavelength for GP and GPP analyzed by HPLC-DAD at (a) 280 nm, (b) 320 nm and (c) 360 nm and (d) 520 nm.

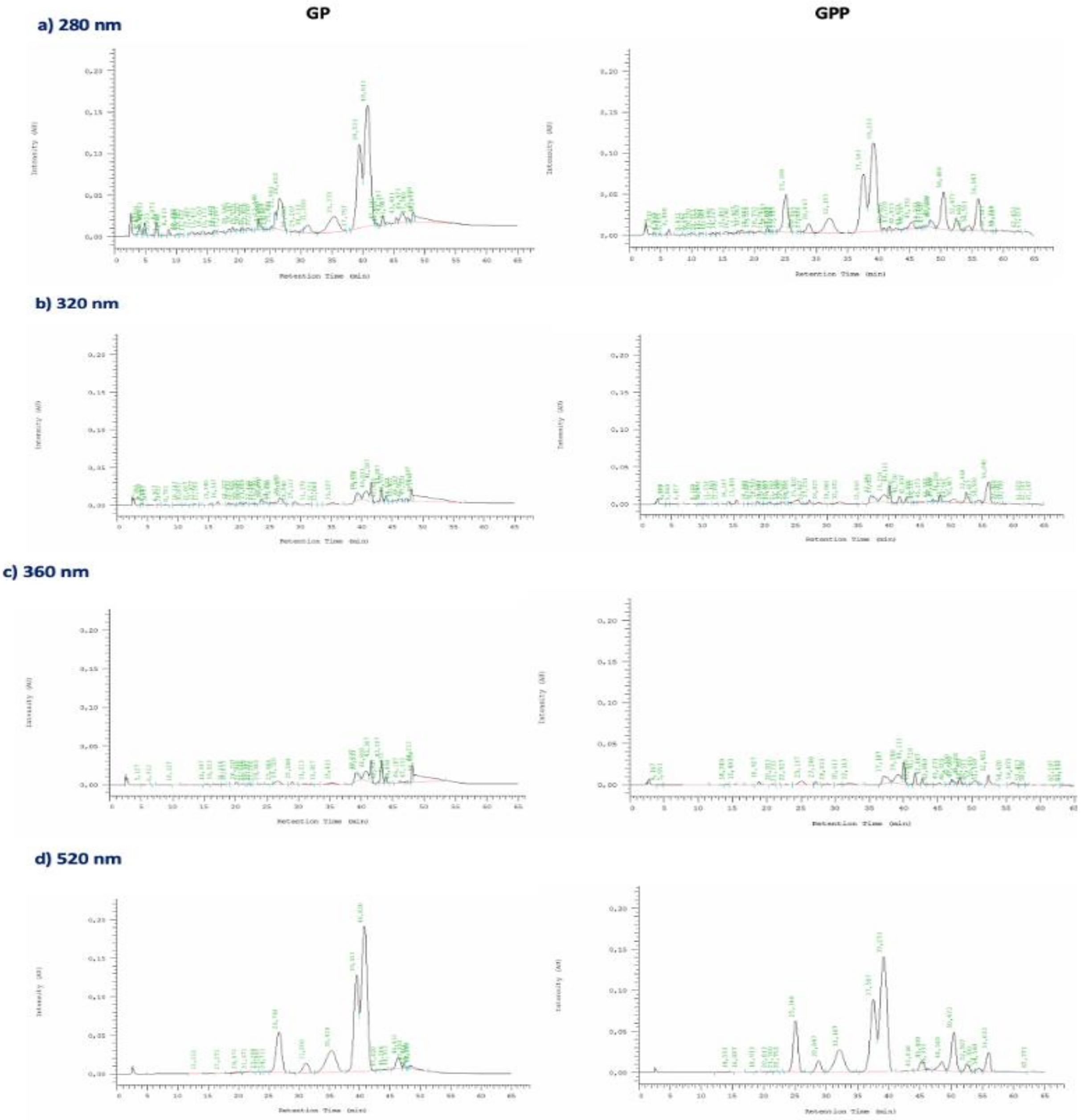

Table S1. Summary of conditions of the preparation of spray-drying

Conditions used	SD
**Inlet air Temperature (°C)**	120
**Feed flow rate (mL min-1)**	4
**Wall material content (% w v-1)**	20

SD: Spray-Drying (powder)

Table S2. Mean values for loading capacity, powder recovery and entrapment efficiency of powder based on grape pomace obtained by spray-drying.

Formulation Method1	Loading capacity2	Powder Recovery3	Entrapment Efficiency4
SD	77.06	42.04	41.10

1SD: Spray-Drying (powder); 2Loading capacity was expressed as g total phenolic compounds per 100 g-1 powder; 3Powder Recovery was expressed as g powder per 100 g-1 initial solids; 4Entrapment efficiency was expressed as g total phenolic compounds encapsulated per 100 g-1 phenolic compounds from pomace added.
